# Supplementary material for: The Impact of Peer Educators or Community Health Workers on the Progress of the UNAIDS 90-90-90 Targets in Africa: A Systematic Review and Meta-Analysis Protocol
Source: Int J Environ Res Public Health. 2021 Apr 8;18(8):3917. doi: 10.3390/ijerph18083917 (PMC8068227; doi:10.3390/ijerph18083917)
Supplement: Supplementary file 1 [file ijerph-18-03917-s001.pdf]

ANNEXES

Supplementary material 1: Full searching strategy by databases

*Medline searching strategy**

#	Concept	Search strategy
1	intervention	(“peer educator” or “HIV patients” or “HIV expert patients” or “community health workers” or “health extension workers” or “adherence supporters”).tw.
2	UNAIDS -1	(HIV care” or “HIV care continuum” or “HIV care cascade” or “first 90” or “hiv diagnos*” or “hiv test*” or “hiv infection” or test* or diagnose or diagnosis or diagnoses or diagnosed or diagnosing or diagnostic or sero-diagnosis or serodiagnosis or prevent* or screen* or “voluntary counseling” or “voluntary counselling” or counseling or counselling or vct).tw.
3	UNAIDS -2	(“second 90” or adherence or retention or attrition or compliance or “hiv care” or “antiretroviral therapy care” or “HIV treatment” or “antiretroviral therapy care” or “ART initiation” or “ART linkage” or “hiv care link*” or “linkages to care” or LTFU or discontinuation or defaulting or “loss to follow-up” or “loss to follow up” or “lost to follow-up” or “lost to follow up” or “loss-to-follow-up” or “lost-to-follow-up” or “loss to retention” or “lost to retention” or “treatment initiation” or retention or retain* or attrition or link* or “link to care” or “link to treatment” or “linkage to care” or “linkage to treatment” or “link into care” or “linkage into care” or “linkage into care” or “linkage into treatment” or eligibility or eligible or eligib* or adher* or complian* or comply or complied or noncomplian* or non-complian* or non-adher* or nonadher).tw.
4	UNAIDS -3	(“third 90” or “viral suppression” or “virological failure” or “clinical failure” or “clinical success” or “immunological failure” or “immunological success” or “treatment failure” or “treatment success”).tw.
5		2 or 3 or 4
6	HIV/AIDS and ART	(hiv or ART or “antiretroviral therapy” or “triple therapy” or HAART or AIDS or UNAIDS or “UNAIDS 90-90-90” or “UN targets” or “HIV Infections” or HIV or “human immunodeficiency virus” or “human immuno deficiency virus” or “human immune deficiency virus” or “human immunedeficiency virus” or “acquired immunodeficiency syndrome” or “acquired immuno deficiency syndrome” or “acquired immune deficiency syndrome” or “acquired immunedeficiency syndrome”).tw.
7	Africa	(Africa or “sub-Saharan Africa” or “list of all African countries”)
8		1 and 5 and 6 and 7; Limited by language (English) and year (2003-2020)

*PubMed searching strategy**

#	Concept	Search strategy
1	intervention	(intervention OR solution OR program OR programme OR strateg* OR polic* OR approach OR evaluation OR promot*)
2	UNAIDS -1	(“first 90” OR “hiv diagnos*” OR “hiv test*” OR “hiv infection” OR test* OR diagnose OR diagnosis OR diagnoses OR diagnosed OR diagnosing OR diagnostic OR sero-diagnosis OR serodiagnosis OR prevent* OR screen* OR “voluntary counseling” OR “voluntary counselling” OR counseling OR counselling OR vct)
3	UNAIDS -2	(“second 90” OR adherence OR retention OR attrition OR compliance OR “hiv care” OR “ART linkage” OR “hiv care link*” OR “linkages to care” OR LTFU OR discontinuation OR defaulting OR “loss to follow-up” OR “loss to follow up” OR “lost to follow-up” OR “lost to follow up” OR “loss-to-follow-up” OR “lost-to-follow-up” OR “loss to retention” OR “lost to retention” OR “treatment initiation” OR retention OR retain* OR attrition OR link* OR “link to care” OR “link to treatment” OR “linkage to care” OR “linkage to treatment” OR “link into care” OR “linkage into care” OR “linkage into care” OR “linkage into treatment” OR eligibility OR eligible OR eligib* OR adher* OR complian* OR comply OR complied OR noncomplian* OR non-complian* OR non-adher* OR nonadher)
4	UNAIDS -3	(“third 90” OR “viral suppression” OR “virological failure” OR “clinical failure” OR “clinical success” OR “immunological failure” OR “immunological success” OR “treatment failure” OR “treatment success”)
5		2 OR 3 OR 4
6	HIV/AIDS and ART	(hiv OR ART OR “antiretroviral therapy” OR “triple therapy” OR HAART OR AIDS OR UNAIDS OR “UN targets” OR “HIV Infections” OR HIV OR “human immunodeficiency virus” OR “human immuno deficiency virus” OR “human immune deficiency virus” OR “human immunedeficiency virus” OR “acquired immunodeficiency syndrome” OR “acquired immuno deficiency syndrome” OR “acquired immune deficiency syndrome” OR “acquired immunedeficiency syndrome”)
7	Africa	(Africa OR “sub-Saharan Africa” OR “list of all African countries”)
8		1 AND 5 AND 6 AND 7 NOT Medline[sb]; Limited by language (English) and year (2003-2020)

Web of Science searching strategy

#	Concept	Search strategy
1	intervention	TS= (intervention or solution or program or programme or strateg* or polic* or approach or evaluation or promot*)
2	UNAIDS -1	TS= (“first 90” or “hiv diagnos*” or “hiv test*” or “hiv infection” or test* or diagnose or diagnosis or diagnoses or diagnosed or diagnosing or diagnostic or sero-diagnosis or serodiagnosis or prevent* or screen* or “voluntary counseling” or “voluntary counselling” or counseling or counselling or vct)
3	UNAIDS -2	TS= (“second 90” or adherence or retention or attrition or compliance or “hiv care” or “ART linkage” or “hiv care link*” or “linkages to care” or LTFU or discontinuation or defaulting or “loss to follow-up” or “loss to follow up” or “lost to follow-up” or “lost to follow up” or “loss-to-follow-up” or “lost-to-follow-up” or “loss to retention” or “lost to retention” or “treatment initiation” or retention or retain* or attrition or link* or “link to care” or “link to treatment” or “linkage to care” or “linkage to treatment” or “link into care” or “linkage into care” or “linkage into care” or “linkage into treatment” or eligibility or eligible or eligib* or adher* or complian* or comply or complied or noncomplian* or non-complian* or non-adher* or nonadher)
4	UNAIDS -3	TS= (“third 90” or “viral suppression” or “virological failure” or “clinical failure” or “clinical success” or “immunological failure” or “immunological success” or “treatment failure” or “treatment success”)
5		2 or 3 or 4
6	HIV/AIDS and ART	TS= (hiv or ART or “antiretroviral therapy” or “triple therapy” or HAART or AIDS or UNAIDS or “UN targets” or “HIV Infections” or HIV or “human immunodeficiency virus” or “human immuno deficiency virus” or “human immune deficiency virus” or “human immunedeficiency virus” or “acquired immunodeficiency syndrome” or “acquired immuno deficiency syndrome” or “acquired immune deficiency syndrome” or “acquired immunedeficiency syndrome”)
7	Africa	TS= (Africa or “sub-Saharan Africa” or “list of all African countries”)
8		1 and 5 and 6 and 7; Limited by language (English) and year (2003-2020)

Scopus searching strategy

#	Concept	Search strategy
1	intervention	ALL (intervention OR solution OR program OR programme OR strateg* OR polic* OR approach OR evaluation OR promot*)
2	UNAIDS -1	ALL (“first 90” OR “hiv diagnos*” OR “hiv test*” OR “hiv infection” OR test* OR diagnose OR diagnosis OR diagnoses OR diagnosed OR diagnosing OR diagnostic OR sero-diagnosis OR serodiagnosis OR prevent* OR screen* OR “voluntary counseling” OR “voluntary counselling” OR counseling OR counselling OR vct)
3	UNAIDS -2	ALL (“second 90” OR adherence OR retention OR attrition OR compliance OR “hiv care” OR “ART linkage” OR “hiv care link*” OR “linkages to care” OR LTFU OR discontinuation OR defaulting OR “loss to follow-up” OR “loss to follow up” OR “lost to follow-up” OR “lost to follow up” OR “loss-to-follow-up” OR “lost-to-follow-up” OR “loss to retention” OR “lost to retention” OR “treatment initiation” OR retention OR retain* OR attrition OR link* OR “link to care” OR “link to treatment” OR “linkage to care” OR “linkage to treatment” OR “link into care” OR “linkage into care” OR “linkage into care” OR “linkage into treatment” OR eligibility OR eligible OR eligib* OR adher* OR complian* OR comply OR complied OR noncomplian* OR non-complian* OR non-adher* OR nonadher)
4	UNAIDS -3	ALL (“third 90” OR “viral suppression” OR “virological failure” OR “clinical failure” OR “clinical success” OR “immunological failure” OR “immunological success” OR “treatment failure” OR “treatment success”)
5		2 OR 3 OR 4
6	HIV/AIDS and ART	ALL (hiv OR ART OR “antiretroviral therapy” OR “triple therapy” OR HAART OR AIDS OR UNAIDS OR “UN targets” OR “HIV Infections” OR HIV OR “human immunodeficiency virus” OR “human immuno deficiency virus” OR “human immune deficiency virus” OR “human immunedeficiency virus” OR “acquired immunodeficiency syndrome” OR “acquired immuno deficiency syndrome” OR “acquired immune deficiency syndrome” OR “acquired immunedeficiency syndrome”)
7	Africa	ALL (Africa OR “sub-Saharan Africa” OR “list of all African countries”)
87		1 AND 5 AND 6 AND 7; Limited Subject area medicine/sociology/psychology AND English; LIMITED to English; LIMITED to year (2003-2020)

*CINAHL Searching strategy**

#	Concept	Search strategy
S1	intervention	intervention or solution or program or programme or strateg* or polic* or approach or evaluation or promot*
S2	UNAIDS -1	"first 90" or "hiv diagnos*" or "hiv test*" or "hiv infection" or test* or diagnose or diagnosis or diagnoses or diagnosed or diagnosing or diagnostic or sero-diagnosis or serodiagnosis or prevent* or screen* or "voluntary counseling" or "voluntary counselling" or counseling or counselling or vct
S3	UNAIDS -2	"second 90" or adherence or retention or attrition or compliance or "hiv care" or "ART linkage" or "hiv care link*" or "linkages to care" or LTFU or discontinuation or defaulting or "loss to follow-up" or "loss to follow up" or "lost to follow-up" or "lost to follow up" or "loss-to-follow-up" or "lost-to-follow-up" or "loss to retention" or "lost to retention" or "treatment initiation" or retention or retain* or attrition or link* or "link to care" or "link to treatment" or "linkage to care" or "linkage to treatment" or "link into care" or "linkage into care" or "linkage into care" or "linkage into treatment" or eligibility or eligible or eligib* or adher* or complian* or comply or complied or noncomplian* or non-complian* or non-adher* or nonadher
S4	UNAIDS -3	"third 90" or "viral suppression" or "virological failure" or "clinical failure" or "clinical success" or "immunological failure" or "immunological success" or "treatment failure" or "treatment success"
S5		S2 or S3 or S4
S6	HIV/AIDS and ART	hiv or ART or "antiretroviral therapy" or "triple therapy" or HAART or AIDS or UNAIDS or "UN targets" or "HIV Infections" or HIV or "human immunodeficiency virus" or "human immuno deficiency virus" or "human immune deficiency virus" or "human immunedeficiency virus" or "acquired immunodeficiency syndrome" or "acquired immuno deficiency syndrome" or "acquired immune deficiency syndrome" or "acquired immunedeficiency syndrome"
S7	Africa	Africa or "sub-Saharan Africa" or "list of all African countries"
S8		S1 and S5 and S6 and S7; Limited by language (English) and year (2003-2020)

Supplementary material 2: JBI Critical Appraisal instruments

JBI Critical Appraisal Checklist for Comparable Cohort/ Case Control

Reviewer ________________________ Date ________________________

Author __________________________ Year _______ Record Number _______

	Yes	No	Unclear	Not Applicable
1. Is sample representative of patients in the population as a whole?	☐	☐	☐	☐
2. Are the patients at a similar point in the course of their condition/illness?	☐	☐	☐	☐
3. Has bias been minimised in relation to selection of cases and of controls?	☐	☐	☐	☐
4. Are confounding factors identified and strategies to deal with them stated?	☐	☐	☐	☐
5. Are outcomes assessed using objective criteria?	☐	☐	☐	☐
6. Was follow up carried out over a sufficient time period?	☐	☐	☐	☐
7. Were the outcomes of people who withdrew described and included in the analysis?	☐	☐	☐	☐
8. Were outcomes measured in a reliable way?	☐	☐	☐	☐
9. Was appropriate statistical analysis used?	☐	☐	☐	☐

Overall appraisal: Include ☐ Exclude ☐ Seek further info. ☐

Comments (Including reason for exclusion)

Supplementary material 3: Tool to Assess Risk of Bias in Cohort Studies

Tool to Assess Risk of Bias in Cohort Studies

1. **Was selection of exposed and non-exposed cohorts drawn from the same population?**

Definitely yes (low risk of bias)	Probably yes	Probably no	Definitely no (high risk of bias)

Examples of low risk of bias: Exposed and unexposed drawn for same administrative data base of patients presenting at same points of care over the same time frame

Examples of high risk of bias: exposed and unexposed presenting to different points of care or over a different time frame

2. **Can we be confident in the assessment of exposure?**

Definitely yes (low risk of bias)	Probably yes	Probably no	Definitely no (high risk of bias)

Examples of low risk of bias: Secure record [e.g. surgical records, pharmacy records]; Repeated interview or other ascertainment asking about current use/exposure

Examples of higher risk of bias: Structured interview at a single point in time; Written self report; Individuals who are asked to retrospectively confirm their exposure status may be subject to recall bias – less likely to recall an exposure if they have not developed an adverse outcome, and more likely to recall an exposure (whether an exposure occurred or not) if they have developed an adverse outcome.

Examples of high risk of bias: uncertain how exposure information obtained

3. **Can we be confident that the outcome of interest was not present at start of study**

Definitely yes (low risk of bias)	Probably yes	Probably no	Definitely no (high risk of bias)

4. **Did the study match exposed and unexposed for all variables that are associated with the outcome of interest or did the statistical analysis adjust for these prognostic variables?**

Definitely yes (low risk of bias)	Mostly yes	Mostly no	Definitely no (high risk of bias)

Examples of low risk of bias: comprehensive matching or adjustment for all plausible prognostic variables

Examples of higher risk of bias: matching or adjustment for most plausible prognostic variables

Examples of high risk of bias: matching or adjustment for a minority of plausible prognostic variables, or no matching or adjustment at all. Statements of no differences between groups or that differences were not statistically significant are not sufficient for establishing comparability.

5. **Can we be confident in the assessment of the presence or absence of prognostic factors?**

Definitely yes (low risk of bias)	Probably yes	Probably no	Definitely no (high risk of bias)

Examples of low risk of bias: Interview of all participants; self-completed survey from all participants; review of charts with reproducibility demonstrated; from data base with documentation of accuracy of abstraction of prognostic data

Examples of higher risk of bias: Chart review without demonstration of reproducibility; data base with uncertain quality of abstraction of prognostic information

Examples of high risk of bias: Prognostic information from data base with no available documentation of quality of abstraction of prognostic variables

6. Can we be confident in the assessment of outcome?

Definitely yes (low risk of bias)	Probably yes	Probably no	Definitely no (high risk of bias)

Examples of low risk of bias: Independent blind assessment; Record linkage; For some outcomes (e.g. fractured hip), reference to the medical record is sufficient to satisfy the requirement for confirmation of the fracture.

Examples of higher risk of bias: Independent assessment unblinded; self-report; For some outcomes (e.g. vertebral fracture where reference to x-rays would be required) reference to the medical record would not be adequate outcomes.

Examples of high risk of bias: uncertain (no description)

7. Was the follow up of cohorts adequate?

Definitely yes (low risk of bias)	Probably yes	Probably no	Definitely no (high risk of bias)

Examples of low risk of bias: No missing outcome data; Reasons for missing outcome data unlikely to be related to true outcome (for survival data, censoring is unlikely to introduce bias); Missing outcome data balanced in numbers across intervention groups, with similar reasons for missing data across groups; For dichotomous outcome data, the proportion of missing outcomes compared with observed event risk is not enough to have a important impact on the intervention effect estimate; For continuous outcome data, plausible effect size (difference in means or standardized difference in means) among missing outcomes is not large enough to have an important impact on the observed effect size; Missing data have been imputed using appropriate methods.

Examples of high risk of bias: Reason for missing outcome data likely to be related to true outcome, with either imbalance in numbers or reasons for missing data across intervention groups; For dichotomous outcome data, the proportion of missing outcomes compared with observed event risk is enough to induce important bias in intervention effect estimate; For continuous outcome data, plausible effect size (difference in means or standardized difference in means) among missing outcomes is large enough to induce clinically relevant bias in the observed effect size.

8. Were co-Interventions similar between groups?

Definitely yes (low risk of bias)	Probably yes	Probably no	Definitely no (high risk of bias)

Examples of low risk of bias: Most or all relevant co-interventions that might influence the outcome of interest are documented to be similar in the exposed and unexposed.

Examples of high risk of bias: Few or no relevant co-interventions that might influence the outcome of interest are documented to be similar in the exposed and unexposed.

Supplementary material 4: JBI Data extraction instruments

JBI Data Extraction Form for Experimental / Observational Studies

Reviewer _______________________ Date _______________________

Author _______________________ Year _______________________

Journal _______________________ Record Number _______________________

Study Method

RCT	☐	Quasi-RCT	☐	Longitudinal	☐
Retrospective	☐	Observational	☐	Other	☐

Participants

Setting _______________________

Population _______________________

Sample size

Group A _______________ Group B _______________

Interventions

Intervention A _______________________

Intervention B _______________________

Authors Conclusions:

Reviewers Conclusions:

Study results

Dichotomous data

Outcome	Intervention ( )<br>number / total number	Intervention ( )<br>number / total number

Continuous data

Outcome	Intervention ( )<br>number / total number	Intervention ( )<br>number / total number

Supplementary material 5: PRISMA-P (Preferred Reporting Items for Systematic review and Meta-Analysis Protocols) 2015 checklist

Section and topic	Item No	Checklist item
**ADMINISTRATIVE INFORMATION**		
Title:		
Identification	1a	Identify the report as a protocol of a systematic review: 1
Update	1b	If the protocol is for an update of a previous systematic review, identify as such NA
Registration	2	If registered, provide the name of the registry (such as PROSPERO) and registration number: 2 and 5, CRD42019135135
Authors:		
Contact	3a	Provide name, institutional affiliation, e-mail address of all protocol authors; provide physical mailing address of corresponding author 1
Contributions	3b	Describe contributions of protocol authors and identify the guarantor of the review 10
Amendments	4	If the protocol represents an amendment of a previously completed or published protocol, identify as such and list changes; otherwise, state plan for documenting important protocol amendments NA
Support:		
Sources	5a	Indicate sources of financial or other support for the review 10
Sponsor	5b	Provide name for the review funder and/or sponsor 10
Role of sponsor or funder	5c	Describe roles of funder(s), sponsor(s), and/or institution(s), if any, in developing the protocol 10
**INTRODUCTION**		
Rationale	6	Describe the rationale for the review in the context of what is already known 3-5
Objectives	7	Provide an explicit statement of the question(s) the review will address with reference to participants, interventions, comparators, and outcomes (PICO) 5-6
**METHODS**		
Eligibility criteria	8	Specify the study characteristics (such as PICO, study design, setting, time frame) and report characteristics (such as years considered, language, publication status) to be used as criteria for eligibility for the review 5-6
Information sources	9	Describe all intended information sources (such as electronic databases, contact with study authors, trial registers or other grey literature sources) with planned dates of coverage 5-7

Search strategy	10	Present draft of search strategy to be used for at least one electronic database, including planned limits, such that it could be repeated 7
Study records:		
Data management	11a	Describe the mechanism(s) that will be used to manage records and data throughout the review 7-8
Selection process	11b	State the process that will be used for selecting studies (such as two independent reviewers) through each phase of the review (that is, screening, eligibility and inclusion in meta-analysis) 7-8
Data collection process	11c	Describe planned method of extracting data from reports (such as piloting forms, done independently, in duplicate), any processes for obtaining and confirming data from investigators 8
Data items	12	List and define all variables for which data will be sought (such as PICO items, funding sources), any pre-planned data assumptions and simplifications 6
Outcomes and prioritization	13	List and define all outcomes for which data will be sought, including prioritization of main and additional outcomes, with rationale 6
Risk of bias in individual studies	14	Describe anticipated methods for assessing risk of bias of individual studies, including whether this will be done at the outcome or study level, or both; state how this information will be used in data synthesis 7-8
Data synthesis	15a	Describe criteria under which study data will be quantitatively synthesised 8-9
	15b	If data are appropriate for quantitative synthesis, describe planned summary measures, methods of handling data and methods of combining data from studies, including any planned exploration of consistency (such as I^2, Kendall's τ) 8-9
	15c	Describe any proposed additional analyses (such as sensitivity or subgroup analyses, meta-regression) 8-9
	15d	If quantitative synthesis is not appropriate, describe the type of summary planned 8
Meta-bias(es)	16	Specify any planned assessment of meta-biases (such as publication bias across studies, selective reporting within studies)9
Confidence in cumulative evidence	17	Describe how the strength of the body of evidence will be assessed (such as GRADE) 9

*** It is strongly recommended that this checklist be read in conjunction with the PRISMA-P Explanation and Elaboration (cite when available) for important clarification on the items. Amendments to a review protocol should be tracked and dated.** **The copyright for PRISMA-P (including checklist) is held by the PRISMA-P Group and is distributed under a Creative Commons Attribution Licence 4.0.**

From: Shamseer L, Moher D, Clarke M, Ghersi D, Liberati A, Petticrew M, Shekelle P, Stewart L, PRISMA-P Group. Preferred reporting items for systematic review and meta-analysis protocols (PRISMA-P) 2015: elaboration and explanation. BMJ. 2015 Jan 2;349(jan02 1): g7647.
